# Supplementary material for: Using a Bayesian network to classify time to return to sport based on football injury epidemiological data
Source: PLoS One. 2025 Mar 20;20(3):e0314184. doi: 10.1371/journal.pone.0314184 (PMC11925455; doi:10.1371/journal.pone.0314184)
Supplement: S1 Fig — (PDF) [file pone.0314184.s001.pdf]

S1 Figure. Structure of the BN model

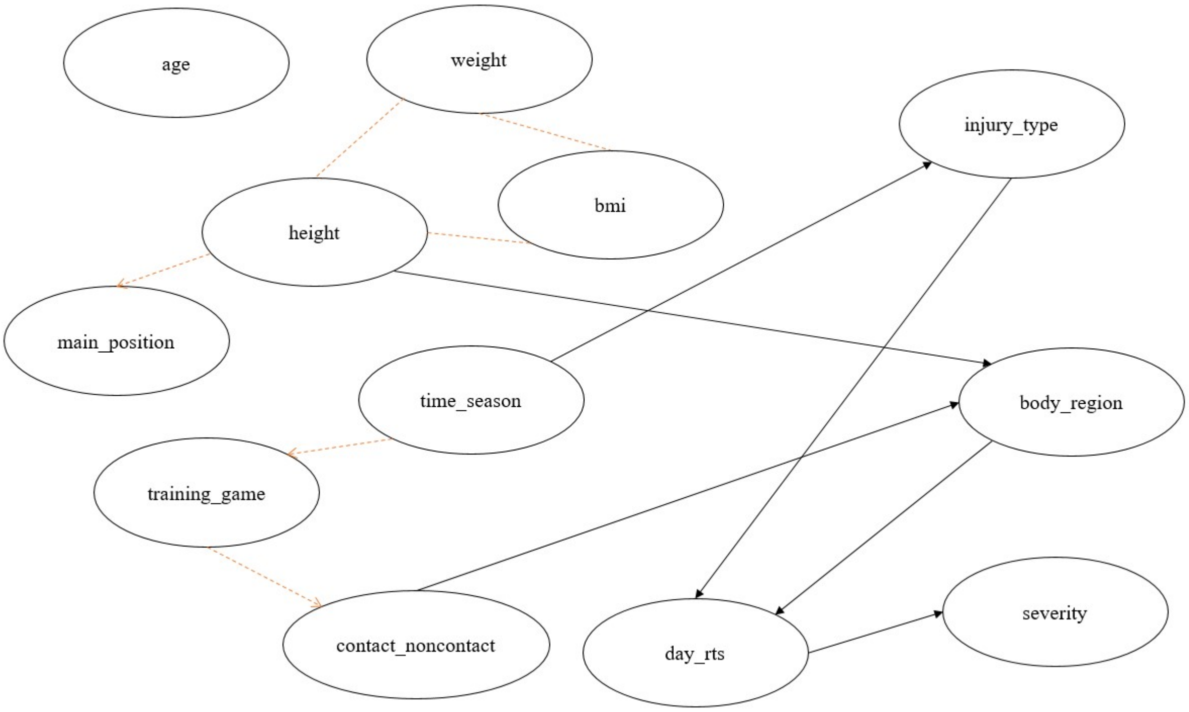

Legend:

Black arrows: directed arcs from data

Dashed lines: expert-elicited arcs
