# Supplementary material for: Using a Bayesian network to classify time to return to sport based on football injury epidemiological data
Source: PLoS One. 2025 Mar 20;20(3):e0314184. doi: 10.1371/journal.pone.0314184 (PMC11925455; doi:10.1371/journal.pone.0314184)

S2 Figures. Tornado plots of the BN model

About tornado plots

- Tornado plots show the sensitivity analysis performed in GeNIe software.
- The label represents the observed state, with the length of the color bar representing the magnitude of influence on the model outcome achieved by changing the specified state.
- The diagram shows the most sensitive parameters for a selected state of the target node.
- The tornado plot displays the variables on the y-axis, with the magnitude of their effect on the outcome represented by horizontal bars. The bars are arranged in descending order based on the magnitude of the effect.
- The colour of the bar indicates the direction of the change in the target state, with red representing a negative change and green indicating a positive change.
- The bar shows the range of changes in the target state as the parameter changes in its range (in this case, 10% of its current value up and 10% down).
- The horizontal axis shows the absolute change in the posterior probability of the state of the target node when each of the parameters changes by that percentage. For example, Figure 1 shows the posterior probability of the node (days_rts = 1-3) when each of the parameters changes by 10%.
- Current parameter value shows the nominal value of the probability in the CPT of the node in question. The probability is identifiable uniquely by the states of the conditioning variables (in Figure 1, body region = thigh, injury_type = muscle_and_tendon).

S2A Figure. Sensitivity for day_rts=rts1_below_3

Current value: 0.216535 Reachable range: [0.21143 .. 0.221641]

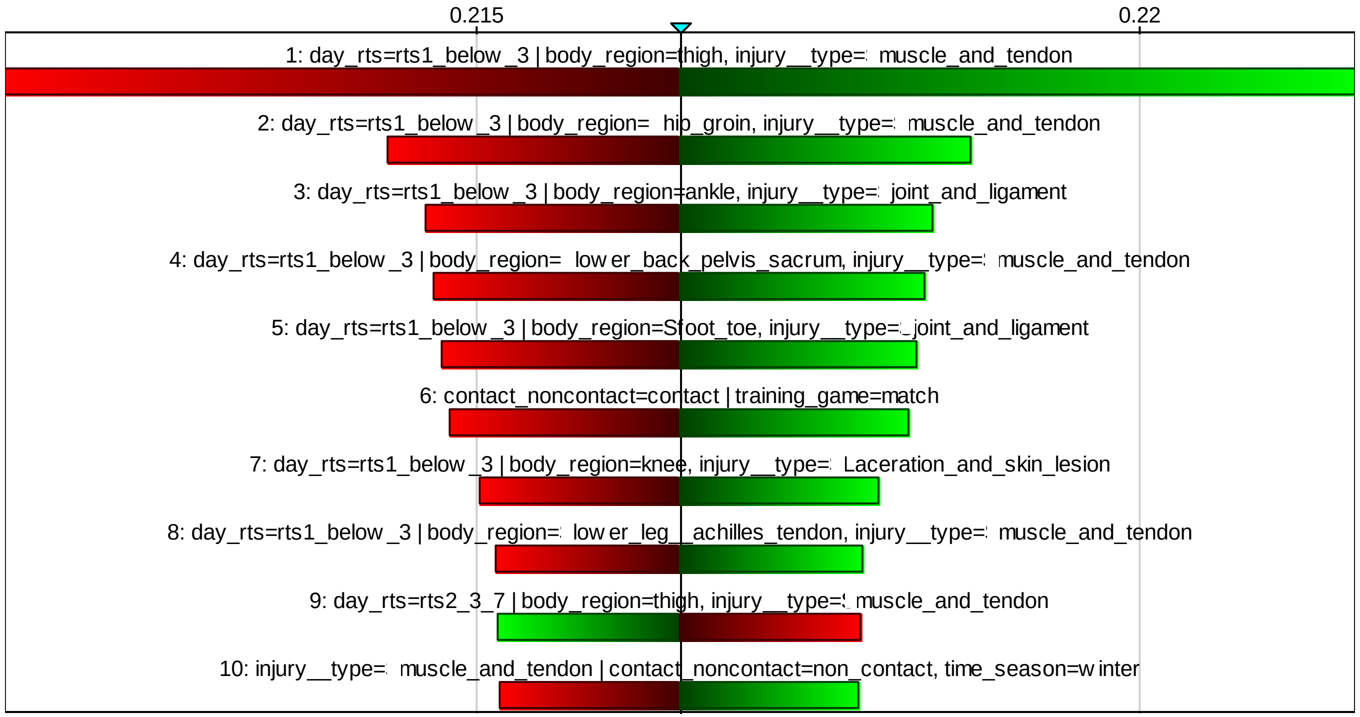

S2B Figure. Sensitivity for day_rts=rts2_3_7

Current value: 0.259196 Reachable range: [0.253844 .. 0.264549]

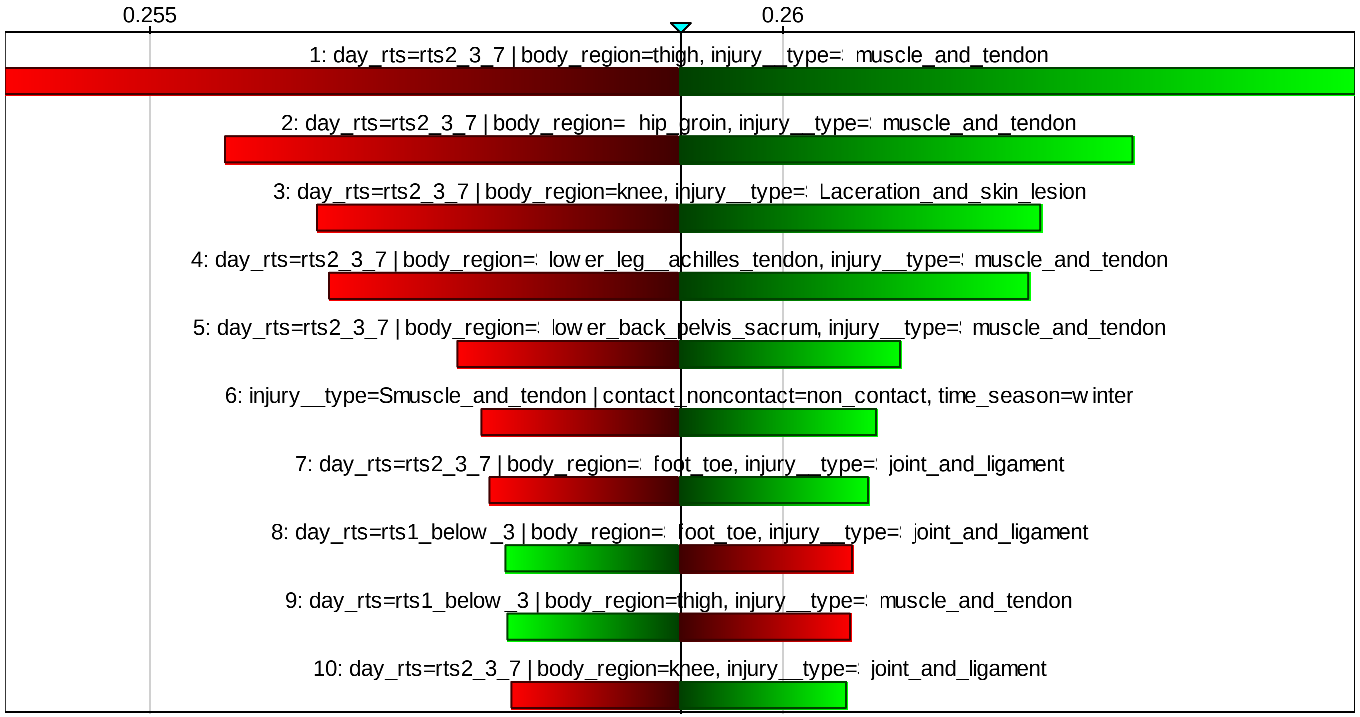

S2C Figure. Sensitivity for day_rts=rts3_7_14

Current value: 0.159266 Reachable range: [0.154737 .. 0.163795]

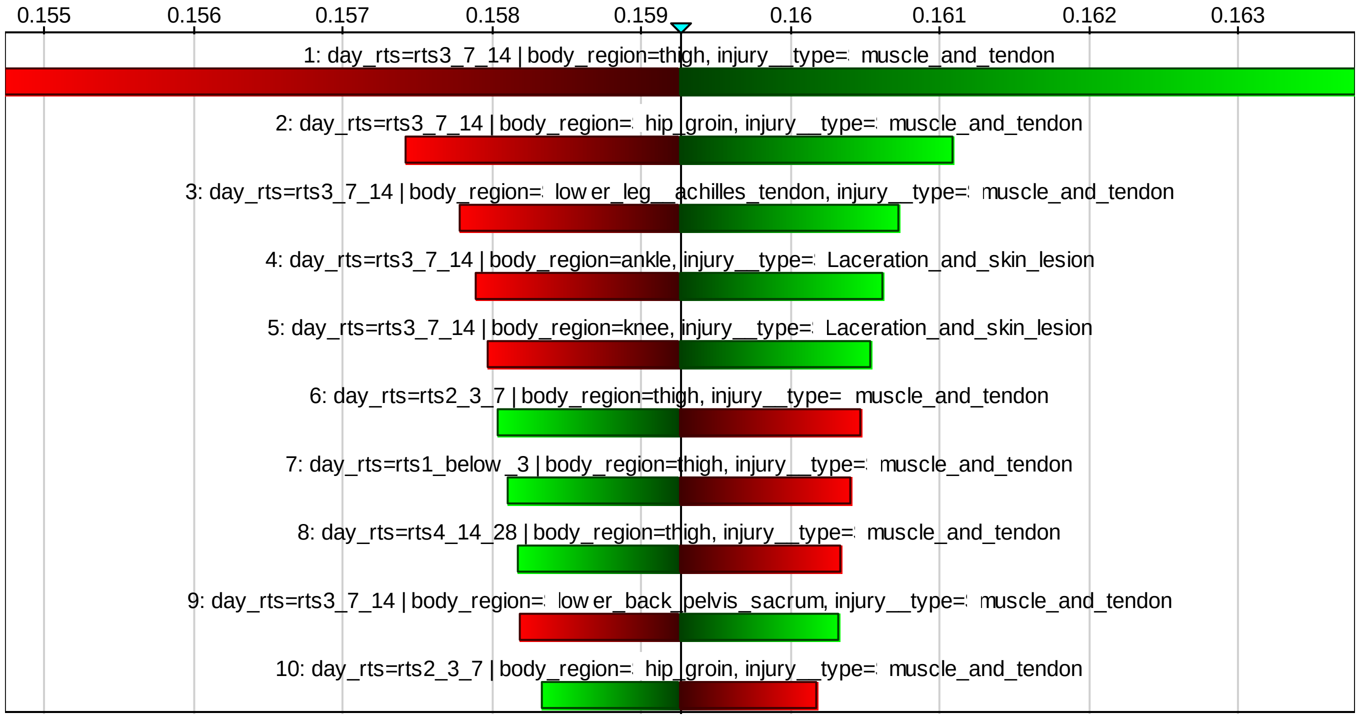

S2D Figure. Sensitivity for day_rts=rts4_14_28

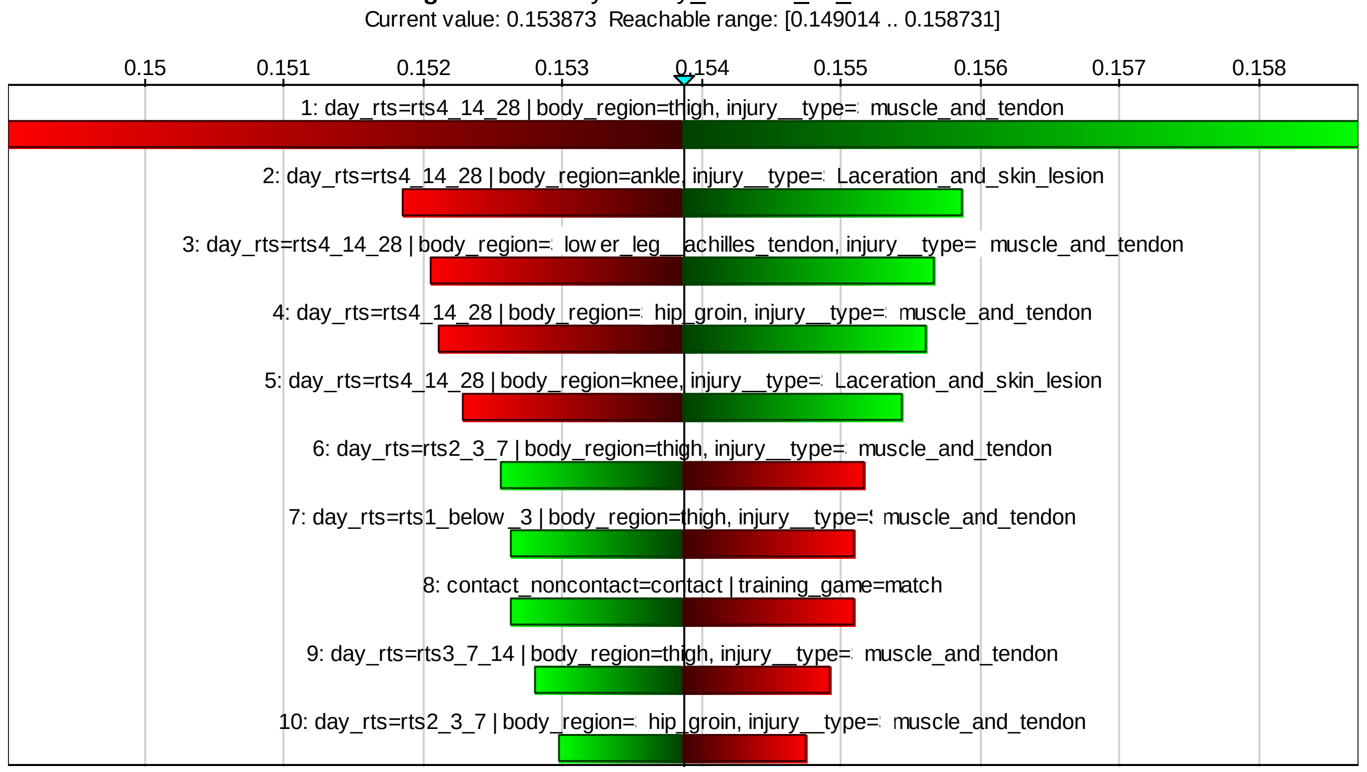

S2E Figure. Sensitivity for day_rts=rts5_28_60

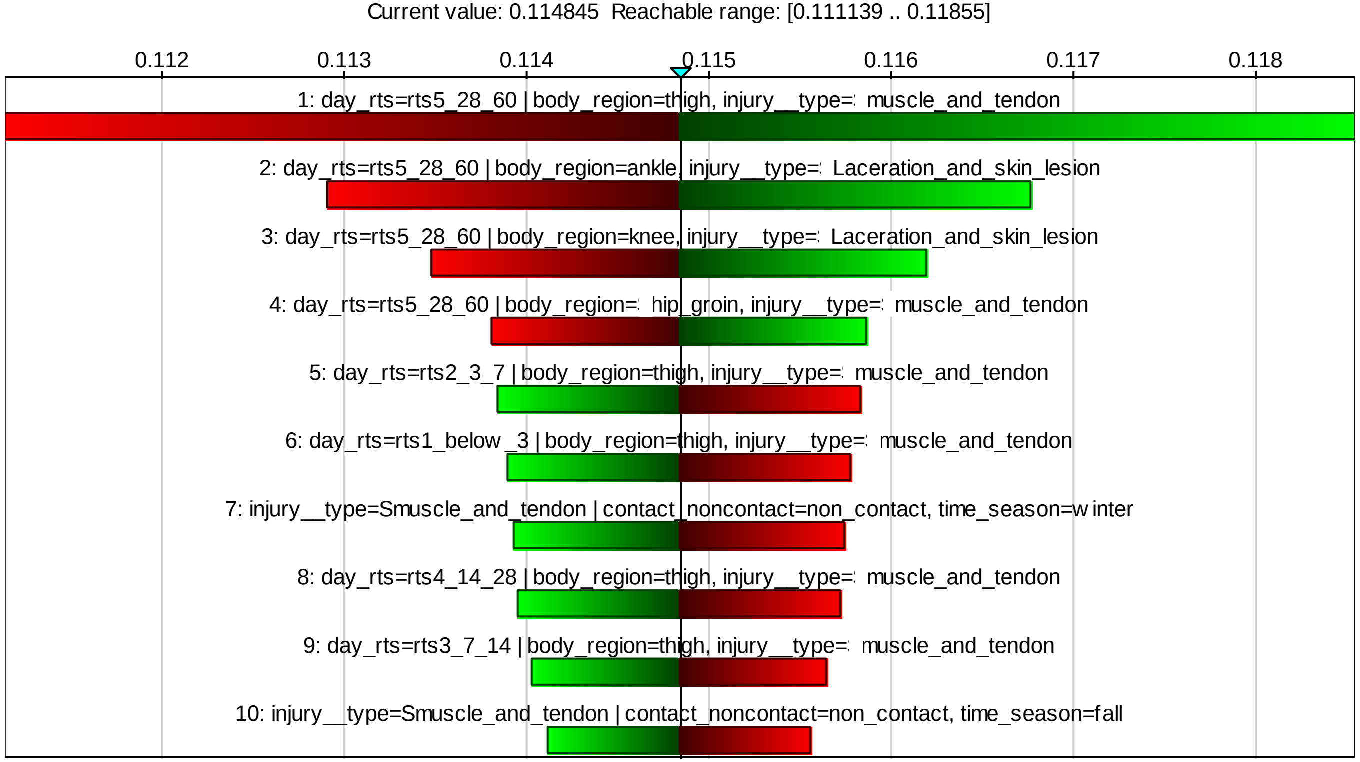

S2F Figure. Sensitivity for day_rts=rts6_60_up

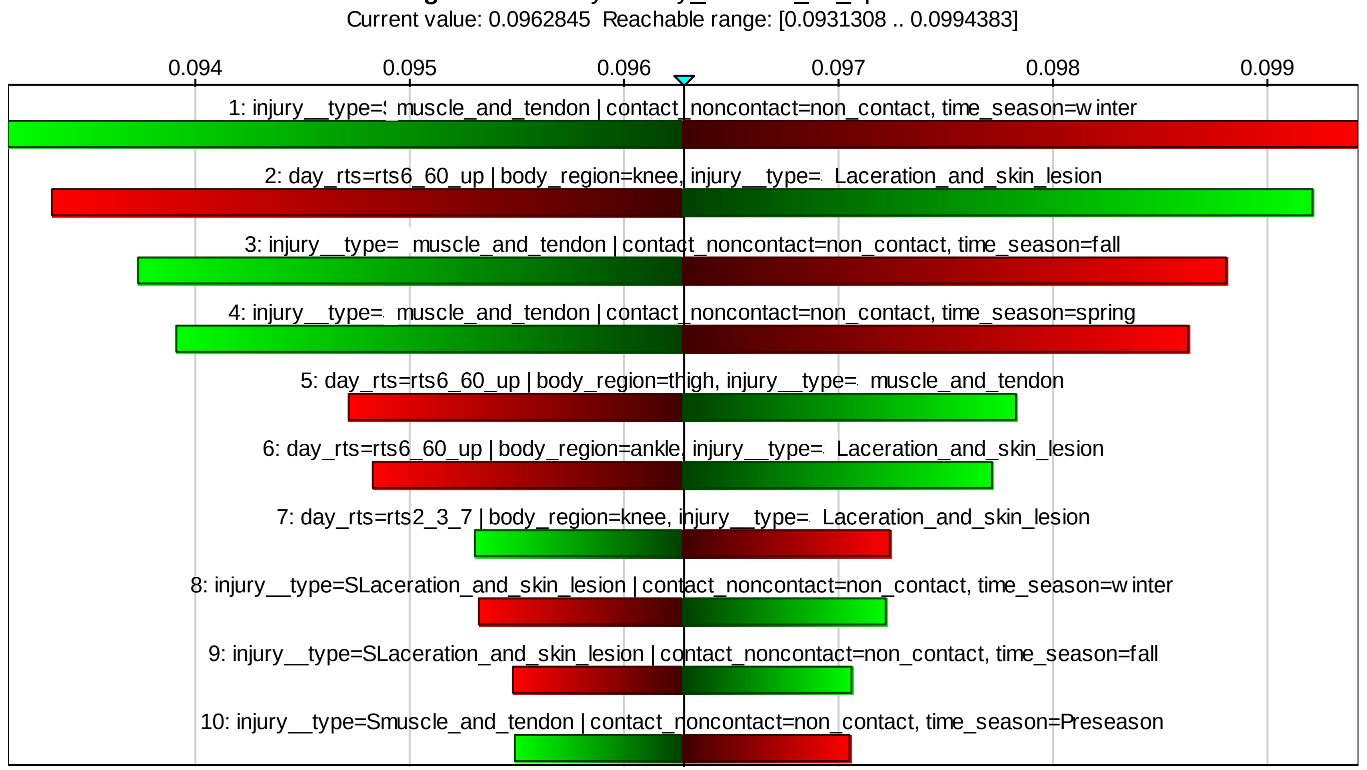

S2G Figure. Sensitivity for severity=minimal

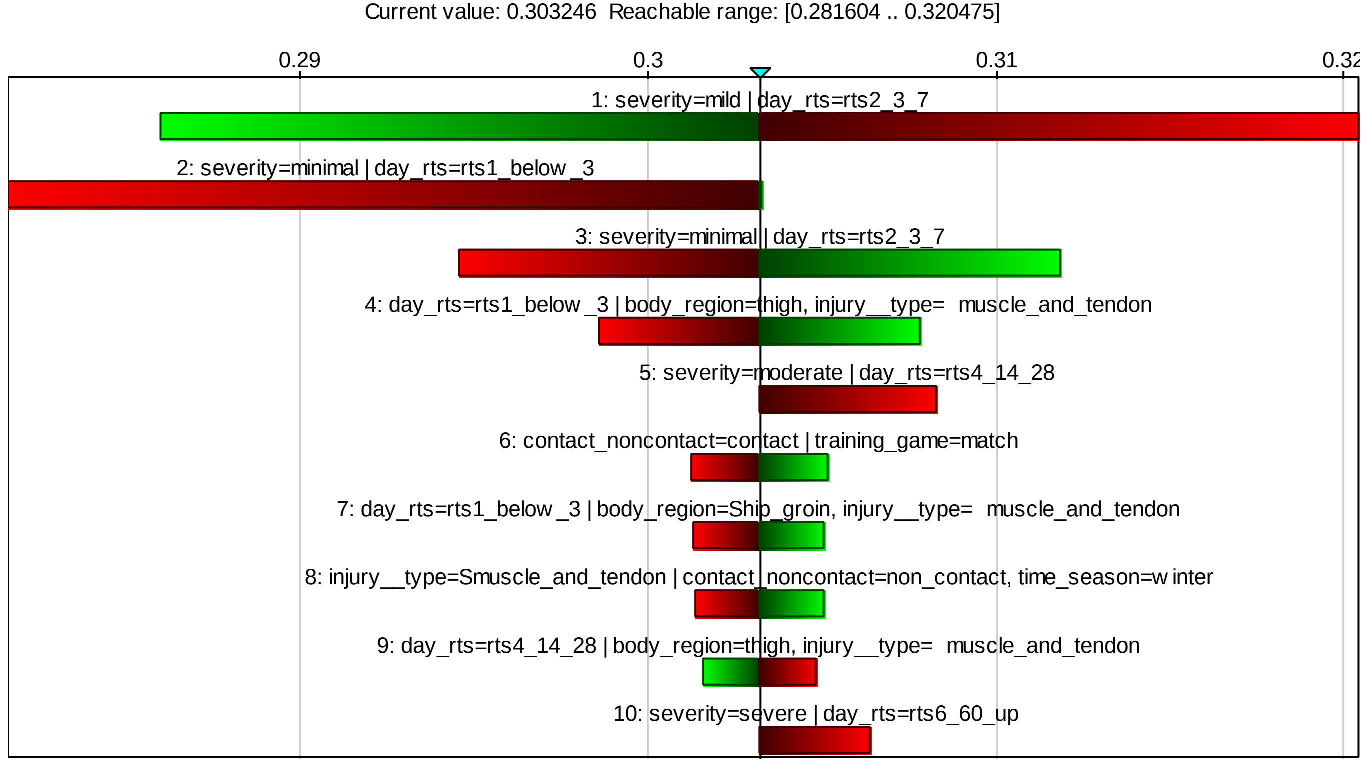

S2H Figure. Sensitivity for severity=mild

Current value: 0.201063 Reachable range: [0.183817 .. 0.218308]

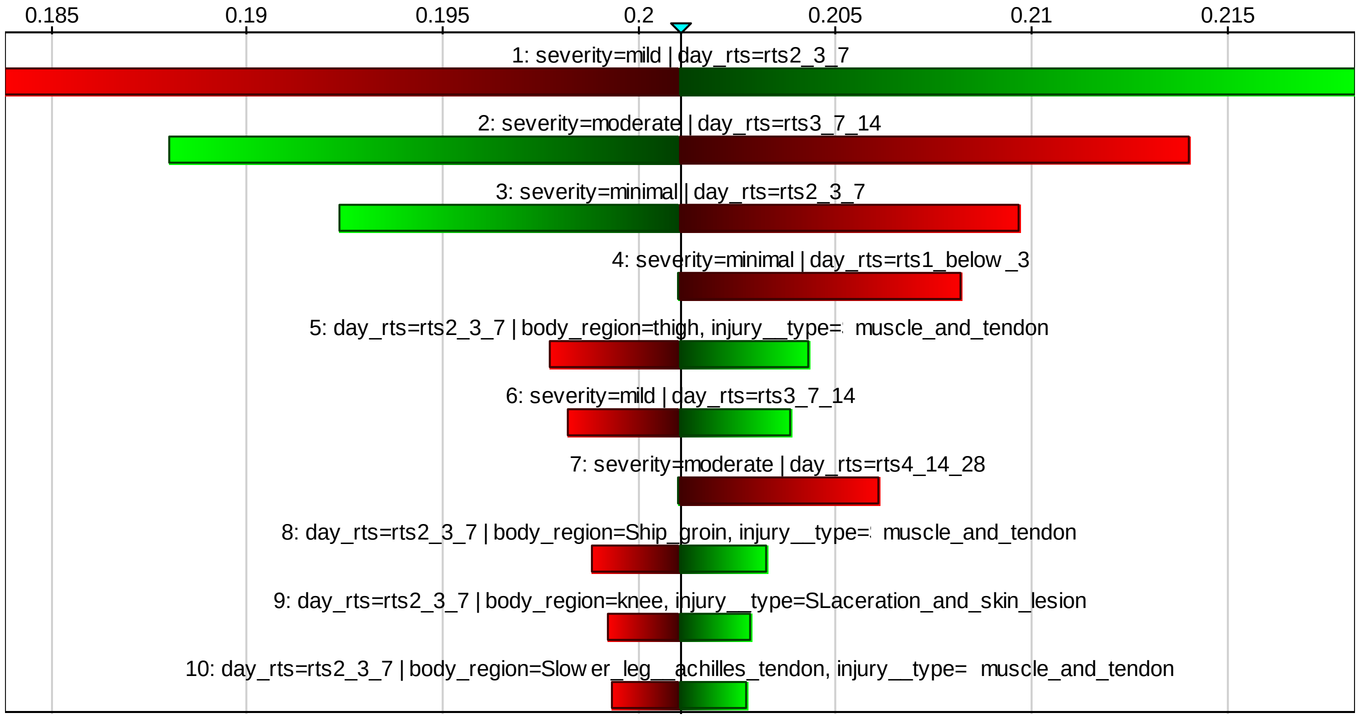

S2I Figure. Sensitivity for severity=moderate

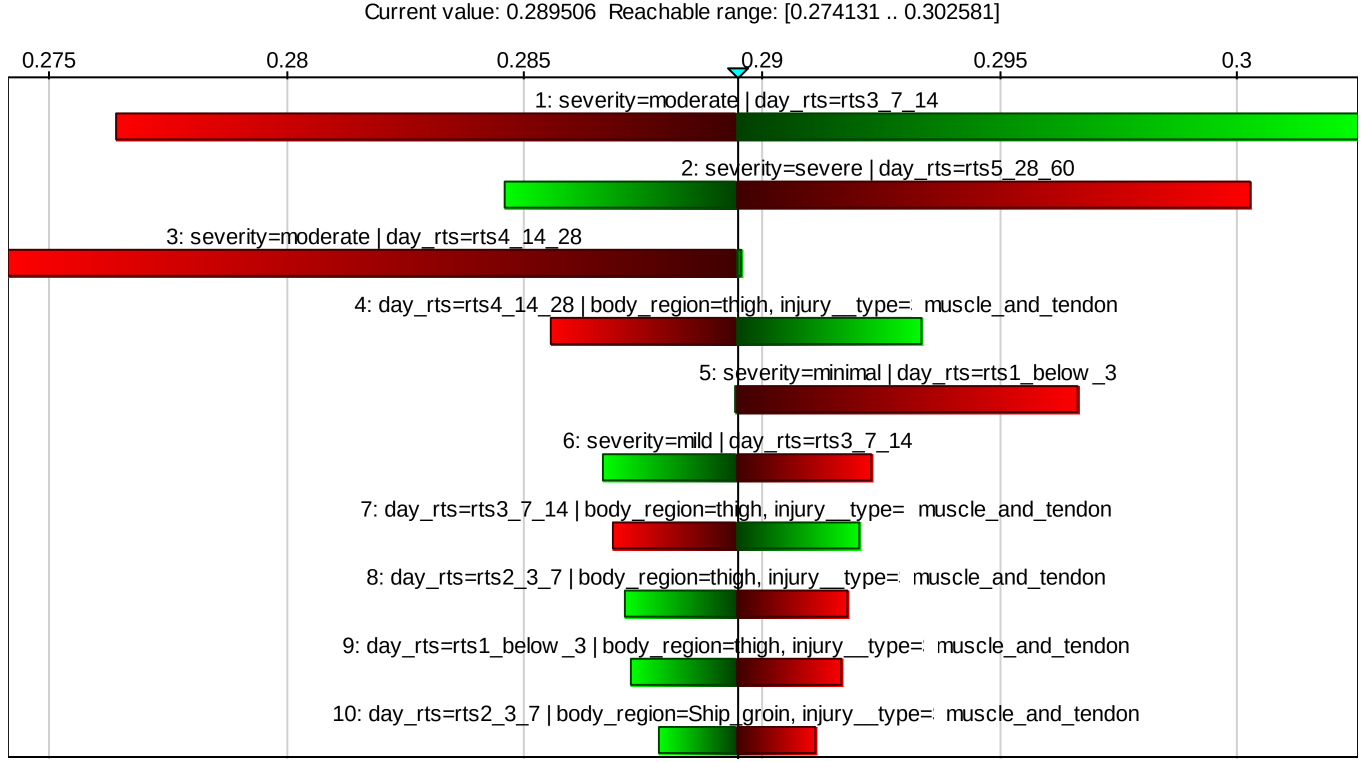

S2J Figure. Sensitivity for severity=severe

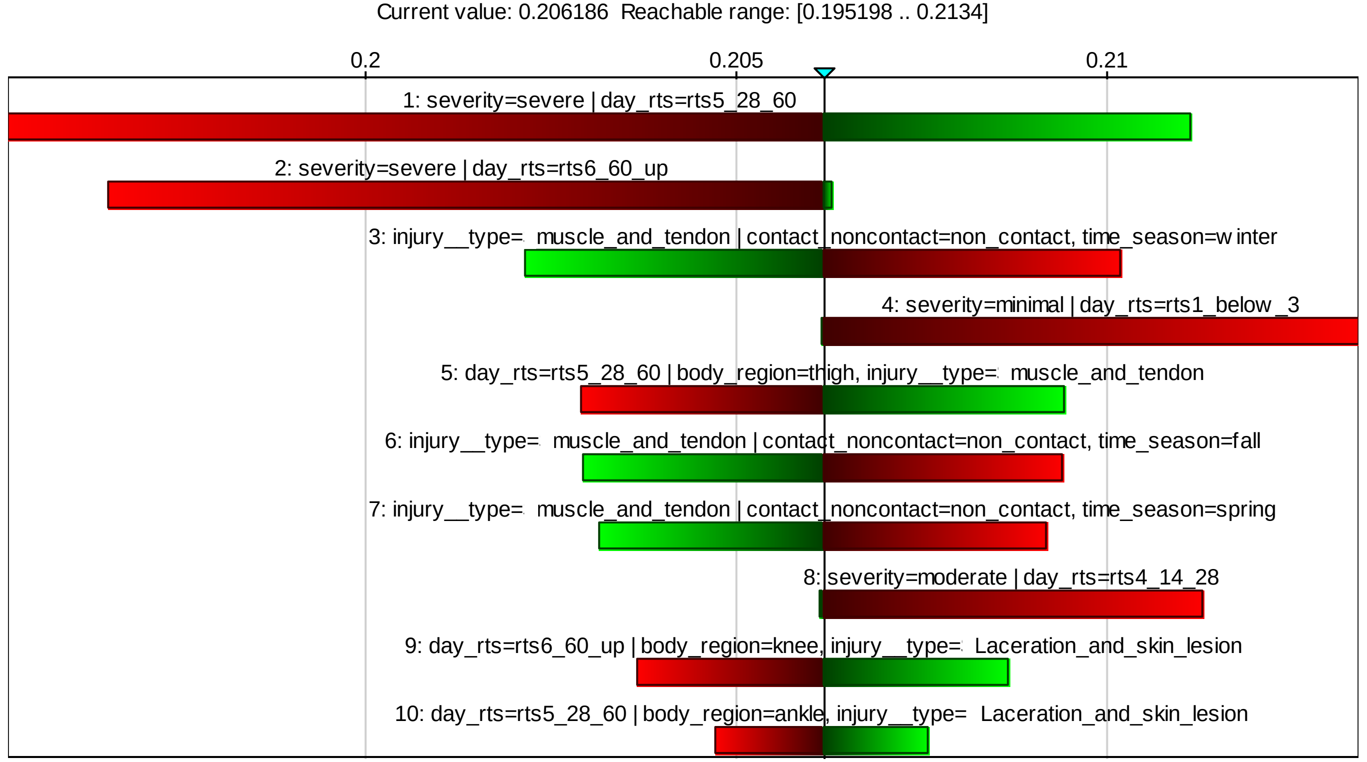

Supplement: S2 Fig — (PDF) [file pone.0314184.s002.pdf]
