# Supplementary material for: Using a Bayesian network to classify time to return to sport based on football injury epidemiological data
Source: PLoS One. 2025 Mar 20;20(3):e0314184. doi: 10.1371/journal.pone.0314184 (PMC11925455; doi:10.1371/journal.pone.0314184)
Supplement: S1 Table — (PDF) [file pone.0314184.s003.pdf]

S1 Table. The demographics and the main playing positions of injured players

Demographics	Average (SD)
**Age (years)**	25.4 ± 3.9
**Height (cm)**	183 ± 6
**Weight (kg)**	78.4 ± 6.8

Playing position	Number of players (n)
**Attacker**	1194
**Midfielder**	2508
**Defender**	2130
**Goalkeeper**	311
