# Supplementary material for: Using a Bayesian network to classify time to return to sport based on football injury epidemiological data
Source: PLoS One. 2025 Mar 20;20(3):e0314184. doi: 10.1371/journal.pone.0314184 (PMC11925455; doi:10.1371/journal.pone.0314184)
Supplement: S2 Table — (PDF) [file pone.0314184.s004.pdf]

**S2 Table. Detail breakdown of injury**

| | | | |
|---|---|---|---|
| **Contact or non-contact** | | | |
| contact | 2092 | | |
| non_contact | 4051 | Total | 6143 |
| | | | |
| **Event** | | | |
| match | 2694 | | |
| training | 3449 | Total | 6143 |
| | | | |
| **Body region** | | | |
| abdomen | 40 | | |
| ankle | 846 | | |
| elbow | 16 | | |
| foot/toe | 367 | | |
| hand/finger/thumb | 41 | | |
| head/face | 226 | | |
| hip/groin | 720 | | |
| knee | 1024 | | |
| lower back/pelvis/sacrum | 425 | | |
| lower leg/ Achilles tendon | 626 | | |
| neck/cervical spine | 59 | | |
| shoulder/clavicular | 120 | | |
| sternum/ribs/upper back | 73 | | |
| thigh | 1554 | | |
| upper arm | 1 | | |
| wrist/forearm | 5 | Total | 6143 |
| | | | |
| **Type of injuries** | | | |
| fracture/bone stress | 300 | | |
| hematoma/contusion/bruise | 849 | | |
| joint and ligament | 1406 | | |
| laceration and skin lesion | 78 | | |
| muscle and tendon | 3339 | | |
| nervous system | 162 | | |
| other injuries | 9 | Total | 6143 |
| | | | |
| **Time of the season** | | | |
| Preseason (Jun-Jul) | 540 | | |
| Spring (Mar-May) | 1618 | | |
| Fall (Aug-Oct) | 1788 | | |
| Winter (Nov-Feb) | 2197 | Total | 6143 |
| | | | |
| **Severity of injuries** | | | |
| Minimal (1-3 days) | 1930 | | |
| Mild (4-7 days) | 1235 | | |

| | | | |
|---|---|---|---|
| Moderate (8-28days) | 1773 | | |
| Severe (>28days) | 1205 | Total | 6143 |
| | | | |
| **Days of absence** | | | |
| 1-3 | 1930 | | |
| 4-7 | 1235 | | |
| 8-14 | 935 | | |
| 15-28 | 838 | | |
| 29-60 | 666 | | |
| >60 | 539 | Total | 6143 |
