# Supplementary material for: Using a Bayesian network to classify time to return to sport based on football injury epidemiological data
Source: PLoS One. 2025 Mar 20;20(3):e0314184. doi: 10.1371/journal.pone.0314184 (PMC11925455; doi:10.1371/journal.pone.0314184)
Supplement: S3 Table — (PDF) [file pone.0314184.s005.pdf]

S3 Table. Strength of influence of all arcs in the model

Parent	Child	Average	Maximum	Weighted
body_region	day_rts	0.204	0.753	0.204
contact_noncontact	injury_ type	0.444	0.461	0.444
contact_noncontact	body_region	0.400	0.840	0.400
day_rts	severity	0.792	0.999	0.792
height	bmi	0.407	0.786	0.407
height	main_position	0.237	0.347	0.237
height	body_region	0.186	0.550	0.186
injury_ type	body_region	0.492	0.815	0.492
injury_ type	day_rts	0.221	0.805	0.221
main_position	day_rts	0.159	0.805	0.159
time_season	training_game	0.096	0.191	0.096
time_season	injury_ type	0.040	0.079	0.040
training_game	contact_noncontact	0.289	0.289	0.289
weight	height	0.503	0.704	0.503
weight	bmi	0.560	0.885	0.560

Note: The strength of influence (and therefore the thickness) of the arcs can be based on three different calculation methods. *Maximum* uses the largest distance between distributions, *Average* takes the plain average over distances, and *Weighted* weighs the distances by the marginal probability of the parent node.

Maximum can give better insight into the strength of influence, as it indicates the largest possible change in the posterior distribution over the child node given a state of the parent node.
