# Supplementary material for: Using a Bayesian network to classify time to return to sport based on football injury epidemiological data
Source: PLoS One. 2025 Mar 20;20(3):e0314184. doi: 10.1371/journal.pone.0314184 (PMC11925455; doi:10.1371/journal.pone.0314184)
Supplement: Attachment — Submitted filename: PlosOne_feedback to reviewers 20250120.docx [file pone.0314184.s007.docx]

Dear Benjamin,

Thanks for the opportunity to revise the manuscript titled “Using a Bayesian network to classify time to return to sport based on football injury epidemiological data.” We appreciate the reviewers’ valuable feedback. Each of the reviewers’ suggestions have been considered carefully. All the reviewers’ comments have been addressed to the best of our ability and can be found in the separate document.

We have also address the additional requirements from journal:

1. We have revise the document to meet PLOS ONE’s style requirement.
2. The Bayesian network described in this manuscript was created using GeNIe Modeler, which has graphical user interface for interactive model building and learning. Therefore, no codes were required.
3. In the cover letter, we have now included this statement "The authors have declared that no competing interests exist.
4. The data used for this study were collected, summarised and verified from various media outlets/databases and therefore include patient privacy. At this level of play (Bundesliga 1), effective annoymisation is also not possible. Therefore sharing the data publicly would compromise patient privacy.
5. We have now included captions for the supporting information files at the end of the manuscript.

“**Supporting information**

S1 Figure. Structure of the BN model

S2 Figures. Tornado plots of the BN model

S1 Table. The demographics and the main playing positions of injured players

S2 Table. Detail breakdown of injury

S3 Table. Strength of influence of all arcs in the model”

We believe these edits and refinements have improved the overall quality of the manuscript and are grateful for the input and feedback. Should any additional edits be requested, we are happy to consider further revisions if deemed most appropriate by the editorial team. Thank you again for considering this manuscript in the *PLOS ONE.*

Sincerely,

Kate, Paul, Anne, Karen and Tim

Authors’ responses to reviewers’ comments in blue

Reviewer #1: Review of the manuscript titled “Using a Bayesian network to classify time to return to sport based on football injury epidemiological data.”

This manuscript provides an example of the possible utility of using Bayesian Network modelling to determine probabilities for time required to return to play for football athletes. The authors are to be congratulated on providing this example of what might be a particularly useful application of Bayesian statistics in applied sports epidemiology.

First of all, I would like to thank for your encouragement and feedback. The following changes have been implemented accordingly.
I have made the following suggestions for editing.

Line 026: Delete the word “of” after the word “factors” and replace with “related to”.

We have revised accordingly: “These variables include intrinsic factors related to the player,…”
Line 028: Insert the words “of time to return to play” after the word “estimation”.

We have revised accordingly: “Providing an individualised estimation of time to return to play is often challenging…”
Line 028: Delete the word “yet” after the word “and”.

We have revised accordingly: “and yet clinical decision support tools are not common in sports medicine.”

Line 029: Delete the words “often rare in the industry” and replace with “not common in sports medicine”.

We have revised accordingly: “and yet clinical decision support tools are not common in sports medicine.”
Line 034: insert the word “a” after the word “applied”.

We have revised accordingly: “…we applied Bayesian network to a set of basic epidemiological data.”
Line 035: Insert “. A” after the word “data”

We have revised accordingly: “A Bayesian network was used as a decision support tool to model the epidemiological data and to integrate clinical data,…”
Line 048: It is reasonable to include a description of the method of sensitivity and specificity analysis as similar to the “producers and users “model from cartography. I suggest that for clarity the terms "sensitivity and specificity" are used throughout the manuscript as these terms will be more familiar to sports medicine practitioners?

We agree that sports medicine practitioners are more familiar with the term “sensitivity” and therefore have changed “producer’s accuracy” to “sensitivity” across the manuscript.

The “users’ accuracy” is not equivalent to “specificity” and therefore we cannot change the term. We have revised the wordings for clarity in lines 293-296, under equation 2:

“The user’s accuracy is calculated by dividing the total number of correctly classified injury severity by the total classified true occurrences of injury of that severity. User's accuracy provides insights into the precision of the model's predictions for each category and how reliable it is from the users’ perspective. (Equation 2).”

Line 061: Delete the word “the”.

We have revised accordingly: “…we suggest further experimenting and developing Bayesian network modelling into a decision-supporting aid.”
Line 061: Insert the word “modelling” after the word “network”.

We have revised accordingly: “…we suggest further experimenting and developing Bayesian network modelling into a decision-supporting aid.”
Line 065: Insert the words “defined as occurring” after the word “is”.

We have revised considered your suggestion but we think that the inclusion of “as occurring” may not be most suitable in this case because here we refer RTP as the time when an injured athlete can return to full unrestricted team training and play without modifications in duration and/ or activities, rather than a process.

Line 065: Insert the words “and play” after the word “training”.

We have revised accordingly: “Return to sport (RTS) is defined as occurring when an injured athlete can return to full unrestricted team training and play without modifications in duration and/ or activities[1-3].”
Line 069: Insert the words “support a graduated progression in the athlete's conditioning program” after the word “to”.

We have revised accordingly: “By having an estimated return date, medical staff can create an individualised rehabilitation plan to support a graduated progression in the athlete's conditioning program to improve the athlete's condition and assist in mitigating re-injury risk.”
Line 069: Delete the word "mitigate” and replace with the words “assist in mitigating”.

We have revised accordingly: “By having an estimated return date, medical staff can create an individualised rehabilitation plan to support a graduated progression in the athlete's conditioning program to improve the athlete's condition and assist in mitigating re-injury risk.”
Line 070: Insert the words “provision of” after the word “The”.

We have revised accordingly: “The provision of predicted timeframe for recovery also helps coaching staff to adjust their game plans and strategies, maximising the team's chances of success.”
Line 070: Insert the words “for recovery” after the word timeframe.

We have revised accordingly: “The provision of predicted timeframe for recovery also helps coaching staff to adjust their game plans and strategies, maximising the team's chances of success.”
Line 077: Insert the word “human physiological” before the word “system”.

We have revised this based on reviewer 2 and your comment:

“Understanding the interactions between the human physiological system's and injury risk is crucial to provide accurate injury prognoses and project an athlete’s likely RTS time range…”
Line 092: Delete the words “Due to the complex systems” and start the sentence at the word “Forecasting”.

We have revised this based on reviewer 2 and your comment:

“Forecasting the time to RTS based on individual characteristics can be challenging for several reasons. First, the Strategic Assessment of Risk and Risk Tolerance framework recommends considering personal characteristics and factors, such as age and playing position[12].”
Line 095: Delete the words “considered” and replace with “included”.

We have revised accordingly: “…are recommended to be included in the Strategic Assessment of Risk and Risk Tolerance framework[13].”
Line 096: Delete the word “these” and replace with “this”.

We have revised accordingly: “quantifying and synthesising this information in the clinical reasoning process can be difficult.”
Line 102: Delete the words “is a lack of tool” and replace with “are no tools”.

We have revised accordingly: “There are no tools to help translate the sheer volume and complexity of the population-level data and tailor them to an individual level.”
Line 103: Delete “on” and replace with “to”.

We have revised accordingly: “There are no tools to help translate the sheer volume and complexity of the population-level data and tailor them to an individual level.”
Line 114: Delete “take charge of” and replace with “undertake the”

We have revised accordingly: “data scientists can undertake the setting up and maintaining the decision support tool,”
Line 129: Insert the word “previously” after the word “been”.

We have revised accordingly: “…it has not been previously used for such a purpose.”

Line 129: Insert the word “a” after the word “such”.

We have revised accordingly: “…it has not been previously used for such a purpose.”
Line 134: Delete the word “In”.

We have revised accordingly: “Figure 1 illustrate how the parent nodes and a child node can be related using graph-based modelling.”
Line 134: There appear to be two Figure 1s? The tornado graph is also labelled as Fig 1 in the supplementary files. I suggest labelling the tornado graph as Fig 4?

Based on the PLOS ONE’s submission guidelines, we have renamed file containing the tornado graphs as S2 Figures, and have labelled each of the tornado graphs as S2A Figures – S2J Figures.
Line 135: Delete the word “we”.\

We have revised accordingly: “Figure 1 illustrate how the parent nodes and a child node can be related using graph-based modelling.”
Line 135: Remove the letter “d” from the word “illustrated”.

We have revised accordingly: “Figure 1 illustrate how the parent nodes and a child node can be related using graph-based modelling.”
Line 140: Insert the word “are” after the word “that”.

We have revised accordingly: “…there are merits of using the discrete Bayesian network that are worth discussing.“
Line 145: Delete the word “discrete” and replace with the word “descriptive”.

We have revised accordingly: “Some may find the descriptive terms are easier to grasp and apply in reasoning.”
Line 147: Insert the word “the” after the word “of”

We have revised accordingly: “…discretisation may help capture the resolution of the data available”
Line 147: Delete the word “relevant” and replace with the words “and it’s relevance”.

We have revised accordingly: “…and it’s relevance to the decision support scenario at hand[23].”
Line 149: Insert the word “also” after the word “it’s”.

We have revised accordingly: “…it's also important to note the disadvantages to discretising continuous variables[24].”
Line 160: Delete the word “brought” and replace with “made”.

We have revised accordingly: “…changes may be made to the node’s marginal probability[26],…”
Line 170: Add an “s” to the word “provide”.

We have revised accordingly: “They combine prior beliefs with the likelihood of the data to provide an updated estimate of the event's probability.”
Line 179: Delete “combination of both of them” and replace with “combinations of these”.

We have revised accordingly: “…,or combinations of these[28]”
Line 189: Delete “use” and replace with “utility”.

We have revised accordingly: “The objective of this study was to demonstrate the use of a Bayesian network and its potential utility in becoming a decision support tool for medical staff for RTS.”
Line 193: Delete the word “a”.

We changed scenarios into scenario instead and kept the word “a”:

“…demonstrate its use with a case scenario (section 4.2).”
Line 253: Insert the words “competition or” after the word “in”.

Sorry we cant locate the word “in” in line 253
Line 259: Insert the word “Author KY established” and delete “helped establish”.

We have revised accordingly: “…Author KY established the relationships (arcs) within the graphical structure”
Line 260: Delete “our” and replace with “the”.

We have revised accordingly: “Notably, the key response variable…”
Line 304: As suggested previously, I suggest consider explaining the similarity between these and sensitivity and specificity and using these terms instead as these will be more easily understood by health readers?

We have addressed this based on your recommendations. Please kindly see feedback made to the earlier comment re line 48.

Line 391: Delete “’ eyes”.

We have revised accordingly: “…we may miss some of the patterns and linkages between variables that were not observed by clinicians”
Line 482: Delete “yield” and replace with “yielded”.

We have revised accordingly: “…the BN yielded similar results to existing clinical systems, its true benefits lie in its ease of use,…”
Line 484: Delete the word “Moreover” and replace with the word “The”.

We have revised accordingly: “The BN's ability to identify the strength of influence allows medical staff to address the variables that significantly impact outcomes,…”
Line 507: Insert the word “The” before the second use of the word “DBN”.

We have revised accordingly: “The DBN is an extension of the BNs and replicates the BN model at discrete points in time (time slices) and captures temporal relationships between the variables.”
Line 508: Insert the word “the” after the word “of”.

We have revised accordingly: “The DBN is an extension of the BNs and replicates the BN model at discrete points in time (time slices) and captures temporal relationships between the variables.”
Line 528: It may be worthwhile extending the discussion to include the possibility of extending the DBN model to examine the possibility of modelling subsequent injury risk?

Thanks for your suggestion. We have add this sentence at the end of the paragraph:

“DBNs have been applied in injury prediction [66], medical diagnosis or prognosis [67-69] and are also a promising avenue for further investigation in subsequent injury risk modelling. Specifically, DBNs may provide medical practitioners with a tool to predict the probability of a subsequent injury to occur given time-series data, such as injury history, recovery time, and players’ physiological state over time. Future work could investigate using Wu et al.(2019)’s approach to analyse scenario-based complex systems [70].”

In the “Supplementary Material Table 3” I suggest that a short explanation of Ave, Max, +Weighted is added to the legend.

We have now added this under S3 Table (previously named as Supplementary Material Table 3)

“Note: The strength of influence (and therefore the thickness) of the arcs can be based on three different calculation methods. *Maximum* uses the largest distance between distributions, *Average* takes the plain average over distances, and *Weighted* weighs the distances by the marginal probability of the parent node.

*Maximum* can give better insight into the strength of influence, as it indicates the largest possible change in the posterior distribution over the child node given a state of the parent node.”

Reviewer #2: General Comments

This paper demonstrated how a Bayesian network model can be used to estimate time to RTS in professional male German footballers. Bayesian networks are very useful in this context, and I commend the authors for using this model, which I think underused in sport, outside of the anti-doping context. Except for the abstract and background, which can be written more clearly, the methods and results were easy to understand. The discussion is relevant, provides a concrete example and does not attempt to overplay the usefulness of the model, which is to augment/guide clinical decision making.

Background: The background can be written more clearly and concisely. Several phrases can be removed without losing meaning. Some paragraphs require some extra context to make them easier to interpret. I’ve provided some examples how various statements can be written more concisely to improve readability.
Thank you for your comments and feedback in improving the clarity of the manuscript. The following changes have been implemented accordingly. We have also reviewed other sections of the manuscript to improve the readability.

Specific Comments

ABSTRACT

The abstract can be written more concisely in several areas without losing meaning. I have provided examples in each section.

Objective:

Original: “This study aims to demonstrate the functions of a Bayesian network by the use of a set of basic epidemiological data.”
Revised: “This study uses epidemiological data to demonstrate a Bayesian Network.”

We have revised accordingly based on your recommendation.
Methods:

Original: “To exemplify the use of Bayesian network in sports medicine, such as providing an individualised estimation time to RTS for individual players, we applied Bayesian network to a set of basic epidemiological data. Bayesian network was used as a decision support tool to model the epidemiological data and to integrate clinical data, non-clinical factors and expert knowledge. Specifically, we used the Bayesian network to capture the interaction between variables in order to classify 1) days to RTS and 2) injury severity (minimal, mild, moderate and severe).”
Revised: “We applied a Bayesian network that integrated clinical, non-clinical factors, and expert knowledge to classify time day to RTS and injury severity (minimal, mild, moderate and severe) for individual players”

We have revised accordingly based on your recommendation.
Original: “Retrospective injury data of 3374 player seasons and 6143 time-loss injuries from seven seasons of the professional German football league (Bundesliga, 2014/2015 through 2020/2021) were collected from public databases and media resources. A total of twelve variables from three main categories (player’s characteristics and anthropometrics, match information and injury information) were included. The key response variables are 1) days to RTS (1-3, 4-7, 8-14, 15-28, 29-60, >60 , and 2) severity (minimal, mild, moderate and severe). As there are more than two categories, producer’s and user’s accuracy was used to reflect the sensitivity and specificity of the model. The producer’s accuracy of the model for days to RTS ranges from 0.24 to 0.97, while for severity categories range from 0.73 to 1.00. The user’s accuracy of the model for days to RTS ranges from 0.52 to 0.83, while for severity categories, it ranges from 0.67 to 1.00.”
Revised: “Retrospective injury data of 3374 player seasons and 6143 time-loss injuries from seven seasons of the professional German football league (Bundesliga, 2014/2015 through 2020/2021) were collected from public databases and media resources. A total of twelve variables from three categories (player’s characteristics and anthropometrics, match information and injury information) were included. The response variables were 1) days to RTS (1-3, 4-7, 8-14, 15-28, 29-60, >60, and 2) injury severity (minimal, mild, moderate, and severe). As there were more than two categories, the producer’s and user’s accuracy were used to reflect the sensitivity and specificity of the model. The producer’s accuracy of the model for days to RTS was 0.24-0.97, while for severity categories it was 0.73-1.00. The user’s accuracy of the model for days to RTS was 0.52-0.83, while for severity categories it was 0.67-1.00.”
We have revised accordingly based on your recommendation.

Conclusion
Original: “The Bayesian network can help to capture different types of data to model the probability of an outcome, such as days to return to sports. In our study, the result from the BN may support coaches and players in predicting days to RTS given an injury, 2) support team planning via assessment of scenarios based on player’s characteristics and injury risk and 3) provide evidence-based support of understanding relationships between factors and RTS. This study shows the key functions and applications of the Bayesian network in RTS, and we suggest further experimenting and developing the Bayesian network into a decision-supporting aid.”
Comment: You have abbreviated Bayesian network as BN for the first time in the conclusion. If you are going to do this, then abbreviate at first use BN from then on.

We have reviewed the abstract and abbreviate Bayesian network as BN when it first appear in the first paragraph:

“This study uses epidemiological data to demonstrate a Bayesian Network (BN).”

Revised: “The Bayesian network can help to integrate different data types to model the probability of an outcome, such as days to return to sport. In our study, the Bayesian network may support coaches and players in predicting days to RTS given an injury, 2) team planning via assessment of scenarios based on players’ characteristics and injury risk, and 3) understanding the relationships between injury risk factors and RTS. This study demonstrates the how a Bayesian network may aid clinical decision making for RTS.”

We have revised accordingly based on your recommendation, and also used BN as the abbreviation.

BACKGROUND

Ln 72-75 – Original: The test below can be written more concisely, as demonstrated:
“There is a growing acknowledgement that athletes operate within a complex system and are subject to the influences of a multitude of variables(4, 5). These factors include previous injury history, current injury, body mass index, playing positions, sociological factors, psychological status, and the nature of the sports event(4).”
Ln 72-75 – Revised: “Athletes operate within a complex system and are influenced by factors such injury history, current injury, body mass index, playing positions, sociological factors, psychological status, and the nature of the sports event(4).”

We have revised accordingly based on your recommendation.
Ln 77-81 – Original: “The human body's complexity and the system's highly intricate nature further complicate the task. Understanding these interactions and their impact on injuries becomes crucial for providing accurate prognoses and projecting a range of the likely RTS time (e.g., there is a 75% chance for the player to return between 4-5 weeks). This could help medical staff and athletes to prepare for both ends of the scenario.”
I think the above an be written more clearly. Suggestion below:
Ln 77-81 – Revised: “Understanding the interactions between body systems and injury risk is crucial to provide accurate injury prognoses and project an athlete’s likely RTS time range (e.g., there is a 75% chance for the player to return between 4-5 weeks). Such RTS projections could help medical staff and athletes to prepare RTS programmes based on both the lower and upper projection limits.”
We have revised accordingly based on your recommendation.

Ln 82-91 – Original: “Large-scale epidemiological studies have made promising strides in providing valuable insights into the expected time to RTS for major injuries in football at the population level(6-9). These studies describe the risk factors for a disease or injury and the extent of the problem (10). Medical staff can use them as a starting point for estimating the time to RTS (11). This approach aligns with the “anchor and adjust” strategy (12), which can be used to optimise predictive accuracy, especially when making decisions in uncertain scenarios (e.g., injury). However, typical epidemiological studies are intended to offer correlations and data based on groups to reflect population-level insights and trends. While this data is valuable for highlighting general patterns and insights, a tool to translate population-level data into individual-level assessments and allow for personalised estimations would be helpful.”
Ln 82-91 – Revised: “Large-scale epidemiological studies have estimated the expected RTS time for major injuries in football at the population level accounting for associated injury risk factors(6-9). Medical staff can then use an “anchor and adjust strategy” to optimise predictive accuracy, thus better estimating RTS times. While typical epidemiological studies aim to estimate population-level effect and trends, tools to translate population-level data into individual-level estimations would be helpful.”
We have revised accordingly based on your recommendation.

Ln 92-96 – Original: “Due to the complex systems, forecasting the time to RTS based on individual characteristics can be challenging. There are several reasons why this process is not straightforward. First, personal characteristics and other factors, such as age and playing position, are recommended to be considered in the Strategic Assessment of Risk and Risk Tolerance framework(13).”
Comment: The phrase “complex systems” is vague. What complex systems are you referring to here. I think this statement can be removed without losing meaning, as in the example below:
Ln 92-96 – Revised: “Forecasting the time to RTS based on individual characteristics can be challenging for several reasons. First, the *Strategic Assessment of Risk and Risk Tolerance Framework* recommends considering personal characteristics and factors, such as age and playing position (13).”
We have revised accordingly based on your recommendation.

Ln 102: Change “There is a lack of tool” to “There is a lack of TOOLS”

Based on reviewer 1 and your comment, we have revised this sentence to:

“There are no tools to help translate the sheer volume and complexity of the population-level data and tailor them to an individual level.”
Ln 107: Add making: “Decision MAKING tool”

We have revised accordingly : “Without an objective and reliable decision making tool specifically designed to provide individualised RTS estimations, medical staff often rely heavily on their clinical experience and population-level epidemiological data.”

Ln 114: Add the “s” to “setting”. “In practical SETTINGS”

We have revised accordingly : “In practical settings,…”

Ln 123: “This includes but is not limited to understanding” should be, “this includes, but is not limited to”. Please add the missing commas.
We have revised accordingly : “This includes, but is not limited…”

METHODS
Ln 140: Add the missing “are” to complete the sentence: “there are merits of using the discrete Bayesian network that ARE worth discussing.”

We have revised accordingly : “there are merits of using the discrete Bayesian network that are worth discussing.”

Ln-193-196: This section is not needed. Your section subheadings provide this information.

This section has been removed.

2.2 – Participants: Was there any consideration for time played? For example, if a player came on for 5 minutes at the end of a game vs. played a full 90 minutes. To some extent, their exposure (time) to injury risk factors during match play is greater and probably needs to be considered. It is also unclear to me from the participants section if training data was collected or not. I presume not if the BN was built from open-source data. Can you state this more clearly?

We understand that the time played (exposure) is related to how likely a player will get injured. However, in this study, we only utilised typical variables that one could find in a football injury epidemiology study on a population level, and did not include the exposure for each individual player in every match and training. If we have access to such data, it may be interesting to include such information in future work. However, we find it difficult to collect such data because clubs are not willing to share that data, even on personal request.

Another issue which we considered was the balance between depth of the data and generalisability of the result. With such depth of the data (i.e. including the training and competition exposure), we may have to be limit the study to one club, which would limit the generalisability of the results. This is not necessary a limitation, but just to share the considerations which we have given to the study design.

DISCUSSION

Ln 413-424: It could be worth discussing how the model can be improved in your final sentence. You have suggested that the contextual information about the extent of the tissue damage and injury location is missing and that is a limitation of the model or reason for reduced accuracy. However, could this information be included in an updated version of the model? If so, I suggest make this clear for the reader as Bayesian network models are quite flexible and can accommodate new information.

Thanks for your suggestion. We have now add this sentence to the end of the paragraph to make this point clear:

“When such information become available, it is possible to incorporate them into the BN modelling as BNs are quite flexible in accommodating new information. However, the BN will have to be trained again with the new data to see if the model’s performance has been improved.”

4.2 – Illustrative Example
This section is nice as it provides a concrete example of how the BN can be used.
I appreciate this is a static example based built on historic data, but the BN is deigned to be used prospectively. Can you model accommodate validation information/model updating? For example, let’s pretend the medical team use the BN to predict the severity and days RTS from a non-contact injury (before assessment by the medical team). The model might come up with a mild injury, 3-7 days until RTS. However, when the medical staff assess the player, they think the injury is severe and the player’s recovery time is much longer, say 28-60 days. Clearly the model is wrong.

The model can accommodate additional information and updating. In this case, we will require a labelled dataset that includes the types of clinical examination, assessment outcomes, and the associated time to return to play. We then train the BN again to see if the model’s performance can be improved. Once the BN modelling is completed, the next time when the medical staff use it, the medical staff can update the BN with the assessment result as a prior and obtain a new prediction.

In the context of a dynamic Bayesian network that can be updated with new data to improve model accuracy, is there any process for the user to refine the model once the clinical outcome is known? Obviously, more data helps, but could some form of user input or reinforcement could be useful to refine the model? For example, if the user can tell the model that it has misclassified the player, then that could be useful to refine future predictions. This is also a consideration in section 4.3.

Similar to above, it is possible for the user to refine the model with new information (e.g., clinical assessment). However, the model has to be trained again to see if the BN’s performance could be improved. The impact on the BN’s performance will depend on how relevant the new information is to the outcome.

If user found that the model has misclassified the player, it is possible to trace the CPTs and node states to find the potential reasons for it. For more complex BNs,/ DBNs, future work could investigate using Wu et al. (2019)’s approach to scenario based complex systems analysis [70]. Future work can use a hybrid approach to improve the BN model. That is, we can first identify the reason for misclassification and then refining the model accordingly.
